# Assessment of Risk of Hereditary Predisposition in Patients With Melanoma and/or Mesothelioma and Renal Neoplasia

**DOI:** 10.1001/jamanetworkopen.2021.32615

**Published:** 2021-11-12

**Authors:** Sounak Gupta, Lori A. Erickson, Christine M. Lohse, Wei Shen, Beth A. Pitel, Shannon M. Knight, Kevin C. Halling, Loren Herrera-Hernandez, Stephen A. Boorjian, R. Houston Thompson, Bradley C. Leibovich, Rafael E. Jimenez, John C. Cheville

**Affiliations:** 1 Department of Laboratory Medicine and Pathology, Mayo Clinic, Rochester, Minnesota; 2 Department of Quantitative Health Sciences, Mayo Clinic, Rochester, Minnesota; 3 Department of Urology, Mayo Clinic, Rochester, Minnesota

## Abstract

**Importance:**

In *BAP1* tumor predisposition syndrome, clear cell renal cell carcinoma (RCC) is frequently associated with melanoma and/or mesothelioma, while germline *MITF* p.E318K alterations are being increasingly reported in melanoma/RCC. Limited data exist on the co-occurrence of melanoma and/or mesothelioma with renal neoplasia and the prevalence of associated germline alterations.

**Objective:**

To assess the frequency of melanoma and/or mesothelioma co-occurring with renal neoplasia using our institutional nephrectomy registry and to determine the prevalence of *BAP1* and *MITF* alterations within this cohort.

**Design, Setting, and Participants:**

In this genetic association study, medical records from 8295 patients from 1970 to 2018, renal neoplasia co-occurring with melanoma and/or mesothelioma within a single institutional nephrectomy registry was reevaluated based on contemporary histopathologic criteria and the medical records were reviewed. Data were analyzed from September 2019 to May 2021.

**Main Outcomes and Measures:**

Identified cases were screened for BAP1 loss using immunohistochemistry; while patients with melanoma and clear cell RCC were screened for *MITF* p.E318K alterations. Tumors from patients with potential germline alterations were analyzed with comprehensive molecular profiling using a 514-gene next generation sequencing panel.

**Results:**

Of a total of 8295 patients, 93 (1.1%; 95% CI, 0.9%-1.4%) had melanoma and/or mesothelioma co-occurring with renal neoplasia (cutaneous melanoma, n = 76; uveal melanoma, n = 11; mesothelioma, n = 6). A total of 69 (74.2%) were male; 24 (25.8%) were female; median age at diagnosis of renal neoplasia was 63 years (IQR, 58-70 years) and the median duration of follow-up was 8.5 years (IQR, 5.0-14.6 years). Two patients with clear cell RCC had germline *BAP1* alterations in the setting of cutaneous melanoma and mesothelioma. Two patients with hybrid oncocytic tumors had biallelic inactivation of *FLCN* in a setting of Birt-Hogg-Dubé (BHD) syndrome associated with uveal melanoma and mesothelioma. Tumor-only screening of clear cell RCC associated with cutaneous (n = 53) and uveal melanoma (n = 6) led to the identification of 1 patient with a likely germline *MITF* p.E318K alteration. After excluding benign renal neoplasia (such as oncocytoma and angiomyolipoma), alterations of *BAP1*, *FLCN*, and *MITF* were identified in 5 of 81 patients (6.2%) with melanoma and/or mesothelioma and renal neoplasia. In contrast to hybrid oncocytic tumors in BHD, no unique genotype-phenotype correlations were seen for clear cell RCC with pathogenic *BAP1/ MITF* alterations and *VHL* loss of function variants. Four of 5 cases (80%) met current National Comprehensive Cancer Network criteria for germline testing based on a combination of age, multifocality, histologic findings, and family history.

**Conclusions and Relevance:**

In this genetic association study, findings support the continued use of these National Comprehensive Cancer Network criteria and suggest more stringent screening may be warranted in this patient population.

## Key Points

**Question:**

What is the frequency of melanoma and/or mesothelioma co-occurring with renal neoplasia and of germline alterations of *BAP1* and *MITF* within this cohort?

**Findings:**

In this genetic association study of medical records of 8295 patients, 93 of 8295 patients (1.1%) who underwent nephrectomy for renal neoplasia were identified with associated melanoma or mesothelioma. Likely germline alterations of *BAP1*, *FLCN*, and *MITF* were identified in 6.2% of cases after excluding benign renal neoplasia (oncocytoma and angiomyolipoma).

**Meaning:**

Results of this study suggest that patients with melanoma and/or mesothelioma and renal neoplasia are at a high risk of a hereditary predisposition syndrome; these results support the continued use of current National Comprehensive Cancer Network guidelines and enhanced testing in this population.

## Teaser

This genetic association study assesses the co-occurrence of melanoma and/or mesothelioma with renal neoplasia, and germline associations with *BAP1* and *MITF* p.E318K alterations in patients who underwent nephrectomy.

## Introduction

Hereditary predisposition syndromes associated with the co-occurrence of melanoma and/or mesothelioma and renal cell carcinoma (RCC) include *BAP1* tumor predisposition syndrome and tumors associated with *MITF* p.E318K alterations.^1,2^

The spectrum of tumor associations in *BAP1* tumor predisposition syndrome has gradually expanded from uveal melanoma and mesothelioma to include early-onset clear cell RCC.^3,4^ For instance, in 1 study,^5^ 13 RCC-affected individuals from 7 families were found to have pathogenic germline alterations involving *BAP1*. In a review of 181 patients with germline *BAP1* variants, the combined frequency of *BAP1* null and missense variants among probands was the highest in uveal melanoma (60 of 181 [33%]), followed by cutaneous melanoma (51 of 181 [28%]) and mesothelioma (41 of 181 [23%]).^1^ In contrast, the incidence of RCC in this cohort was lower (12 of 181 [7%]; 4 of 40, and 8 of 121 probands with missense and null variants, respectively).^1^

The *MITF* p.E318K alteration leads to impaired sumoylation and transcriptional dysregulation and has been associated with both a high nevi count (>200) and increased risk of melanoma.^6,7^ A separate study^2^ showed an odds ratio of 4.78 for melanoma, while the odds ratio for combined melanoma and RCC among carriers of this alteration was as high as 14.46.

Nevertheless, to date, relatively limited data exist on the co-occurrence of melanoma and/or mesothelioma with renal neoplasia and germline associations with *BAP1* and *MITF* p.E318K alterations. Herein, we sought to address this association in our institutional nephrectomy registry.

## Methods

### Patient Specimens

This study was approved by the institutional review board at the Mayo Clinic in Rochester, Minnesota, and the need for informed consent was waived for this study owing to minimal risk to patient welfare. The nephrectomy registry was queried for patients with a renal neoplasm diagnosed between 1970 and 2018 (8295 patients) and a documented history of melanoma and/or mesothelioma. The medical records were accessed for relevant clinicopathologic characteristics including all associated neoplasia, documentation of germline testing, and syndromic associations, if any. Data were analyzed from September 2019 to May 2021. This study followed the Strengthening the Reporting of Genetic Association Studies (STREGA) reporting guideline.

### Histopathologic Review and Immunohistochemistry

All 93 identified cases underwent histopathologic review based on contemporary criteria. In addition, immunohistochemistry (IHC) was performed on whole slide sections using an antibody against BAP1 (clone C-4, 1:50 dilution; Santa Cruz Biotechnology). Screening was performed to identify cases with complete loss of staining in the tumor cells.

### DNA Sequencing

Polymerase chain reaction was performed on genomic DNA extracted from formalin-fixed, paraffin-embedded tumor specimens to screen for the *MITF* p.E318K alteration only for clear cell RCC associated with cutaneous (n = 53) and uveal melanoma (n = 6), performed by a genomics service provider (GENEWIZ; Azenta Life Sciences). Genomic DNA from formalin-fixed, paraffin-embedded tumors of patients with potential germline alterations (n = 5) underwent comprehensive molecular profiling using the clinically validated MayoComplete Solid Tumor Panel that interrogates the coding sequence of 514 genes for single nucleotide variants and small insertions/ deletions.

### Statistical Analysis

This study involved minimal statistical analysis. No statistical software was used and no *P* values have been reported in this study. Continuous features were summarized with medians and IQRs, and categorical features were summarized with frequencies and percentages.

## Results

Registry information from a total of 8295 patients was included in this study. Co-occurrence of melanoma and/or mesothelioma and renal neoplasia was identified for 93 of 8295 patients (1.1%; 95% CI, 0.9%-1.4%): 76 with cutaneous melanoma, 11 with uveal melanoma, and 6 patients with mesothelioma (eFigure in the Supplement). These patients exhibited a male predilection (69 were male [74.2%]; 24 were female [25.8%]), the median age at diagnosis of renal neoplasia was 63 years (IQR, 58-70 years) and the median duration of follow-up was 8.5 years (IQR, 5.0-14.6 years). Clear cell RCC was the most common tumor type (n = 62) (eFigure in the Supplement). Other tumor types included papillary RCC (n = 10), angiomyolipoma (n = 6), oncocytoma (n = 6), chromophobe RCC (n = 3), unclassified RCC (n = 3), hybrid oncocytic tumors (n = 2), and *TFEB*-amplified RCC (n = 1) (eFigure in the Supplement). Of note, both patients with hybrid oncocytic tumors met clinical criteria for BHD syndrome. Herein, we found that the prevalence of *BAP1*, *FLCN*, and *MITF *p.E318K alterations in this cohort was 6.2% (5 of 81 patients, after excluding benign renal neoplasia, such as oncocytoma and angiomyolipoma; 95% CI 2.3%-14.5%) (eFigure in the Supplement). Furthermore, 4 of 5 patients in our study met current National Comprehensive Cancer Network criteria for germline testing based on a combination of age, multifocality, histologic findings, and family history.

### *BAP1* Alterations

Two of 93 patients (2.2%) showed BAP1 loss by IHC in clear cell RCC tumor cells. Germline *BAP1* alterations were documented in the medical record for these 2 patients (Table). The first patient (germline *BAP1* p.K453Rfs*) underwent a radical nephrectomy at 46 years of age for 2 synchronous clear cell RCCs and subsequently developed epithelioid mesothelioma and cholangiocarcinoma. The second patient presented with bilateral metachronous clear cell RCCs and associated neoplasia included cutaneous melanoma and a pancreatic neuroendocrine tumor. Of note, this patient had a strong family history of renal neoplasia in 2 first degree relatives. These clear cell RCCs were characterized by high nucleolar grade, eosinophilic cytoplasmic granules, unusual nuclear pseudo-inclusions and exhibited loss of BAP1 by IHC (Figure, A and B).

**Table.** Germline *BAP1* Alterations Documented in the Medical Record

Case	Age at nephrectomy, y	Sex	Renal neoplasia: histologic findings	Size, cm	WHO grade	pTNM	Associated neoplasia, age at presentation (y) and follow-up	Family history	Germline/somatic alterations in renal tumor
1	Left, radical nephrectomy: 46	Male	Clear cell RCC (n = 2, synchronous)	Tumor1: 2.4; tumor2: 2.0	Tumor1: Gr3; tumor2: Gr4	Tumor1: pT1a, N0, M0; tumor2: pT1a, N0, M0	Epithelioid mesothelioma (48); cholangiocarcinoma (54); DOD (55)	No documented family history of renal neoplasia, melanoma, or mesothelioma	(Germline) *BAP1* p.K453Rfs*; (somatic) *VHL* p.N131Y
2	Right, radical nephrectomy: 59; left, cryoablation: 69	Male	Clear cell RCC (n = 2, bilateral and metachronous)	Tumor1: 6.2; tumor2: 1.6 (imaging)	Tumor1: Gr3; tumor2: Gr2	Tumor1: pT1b, N0, M0; tumor2: NA	Cutaneous melanoma (65); pancreatic neuroendocrine tumor (65); AWOD (71)	Son: clear cell RCC (42); brother: melanoma (53), renal neoplasia (57); nephew: melanoma (22); niece: melanoma (20s)	(Germline) *BAP1* p.L573fs*; (somatic) *VHL* p.N131Tfs28*
3	Left, radical nephrectomy: 61	Male	Clear cell RCC	7.0	Gr3	pT3b, N0, M1	Cutaneous melanoma (65); AWD (71)	No documented family history of renal neoplasia, melanoma, or mesothelioma	(^a^Likely germline) *MITF* p.E318K (VAF: 52%); (^a^likely somatic) *VHL* p.Y185* (VAF: 10%)
4	Left, partial nephrectomy: 54	Male	Hybrid oncocytic tumor (n = 2, synchronous)	Tumor1: 3.0; tumor2: 1.3	NA	NA	BHD: renal neoplasia, emphysema, pneumothorax; malignant (epithelioid) pleural mesothelioma (56); DOD (mesothelioma, 59)	No documented family history of renal neoplasia, melanoma, or mesothelioma	(^a^Likely germline) *FLCN* p.E335* (VAF: 50%); (^a^likely somatic) *FLCN* p.I198fs* (VAF: 36%)
5	Right, radical nephrectomy: 48; left, partial nephrectomy: 48 and 58	Male	Hybrid oncocytic tumor (n = 12, bilateral and metachronous)	(Right) 0.4 to 12.0; (left) 2.1 to 4.2	NA	NA	BHD: renal neoplasia, lid folliculomas, emphysema; uveal melanoma (63; previously reported by Fontcuberta et al^8^); DOD (melanoma, 66)	No documented family history of renal neoplasia, melanoma, or mesothelioma	(Germline) *FLCN* p.H429fs*; (somatic) *FLCN* p.P30fs*

Abbreviations: AWD, alive with disease; AWOD, alive without disease; BHD, Birt-Hogg-Dubé syndrome; DOD, dead of disease; Gr, grade; NA, not applicable; pTNM, pathologic tumor, lymph node, metastasis stage (*American Joint Committee on Cancer, 8th edition*); RCC, renal cell carcinoma; VAF, variant allele frequency; WHO, World Health Organization.

^a^ Tumor only sequencing.

**Figure.**
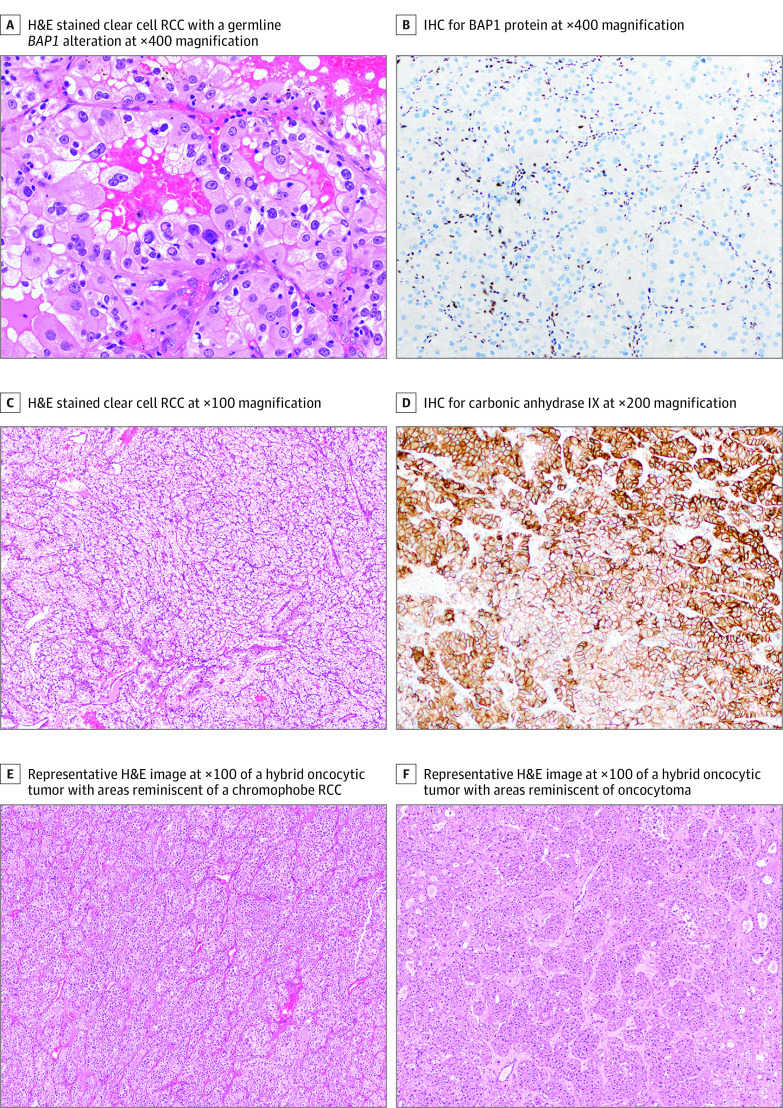
Histopathologic and Immunohistochemistry Imaging A representative hematoxylin-eosin (H&E)–stained image (A) of a clear cell RCC with a germline *BAP1* alteration (p. K453Rfs*, case 1) and corresponding IHC for BAP1 protein is shown (B) at original magnification  ×400. An H&E stained image (C, original magnification ×100) and IHC for carbonic anhydrase IX (CAIX, D, original magnification ×200) is shown for case 3 (*MITF* p. E318K). Representative H&E images of a hybrid oncocytic tumor from case 5 with a germline *FLCN* p.H429fs* alteration has been shown, with areas reminiscent of a chromophobe RCC (E) and oncocytoma (F); images acquired at original ×100 magnification.

### *MITF* p.E318K Alteration

Screening of 59 cases of clear cell RCC (cutaneous melanoma, n = 53; uveal melanoma n = 6) led to the identification of 1 patient with this alteration (Table). This patient underwent a nephrectomy for a clear cell RCC and tumor only sequencing revealed a likely germline *MITF* p.E318K alteration (variant allele frequency (VAF): 52%) with a likely somatic alteration of *VHL* (VAF: 10%). This patient subsequently developed cutaneous melanoma but did not have any relevant family history of cancer. Diffuse positivity for carbonic anhydrase IX was identified in this clear cell RCC, likely because of constitutive signaling by hypoxia inducible factors secondary to *VHL* inactivation (Figure, C and D).

### *FLCN* Alterations

Unexpectedly, 2 patients with Birt-Hogg-Dubé (BHD) syndrome were identified based on review of the medical record (Table). Both patients presented with multiple hybrid oncocytic (renal) tumors (Figure, E and F). The first patient with a likely germline *FLCN* p.E335* alteration (identified on tumor-only sequencing, VAF: 50%) developed mesothelioma, which has been rarely reported in BHD patients.^9^ The second patient (with a germline *FLCN* p. H429fs* alteration) subsequently developed uveal melanoma and this case has been previously reported.^8^

## Discussion

Using a large cohort of patients with renal neoplasia, we found that co-occurrence of melanoma and/or mesothelioma with renal neoplasia was seen in 1.1% of cases. Moreover, we found that the prevalence of *BAP1*, *FLCN*, and *MITF* p.E318K alterations in this cohort was 6.2% (5 of 81 patients, after excluding benign renal neoplasia, such as oncocytoma and angiomyolipoma; 95% CI 2.3%-14.5%). These findings highlight the high risk of a hereditary predisposition syndrome in this patient population.

Although most patients with *BAP1* tumor predisposition syndrome and a renal tumor have presented with clear cell RCC, at least 2 patients with a clear cell RCC and associated non–clear cell RCC (chromophobe RCC and multiple papillary RCC) have been reported.^10^ Of note, loss of BAP1 protein expression by IHC was identified in the chromophobe RCC.^10^ The clear cell RCCs identified in this study had likely somatic alterations involving the *VHL* gene, and although they exhibited some unusual morphologic features, such as the presence of nuclear pseudo-inclusions, our results suggest a lack of unique genotype-phenotype associations for renal neoplasia in *BAP1* tumor predisposition syndrome. For a limited number of patients with *MITF* p.E318K alterations and renal tumors, in which the histologic subtype was specified, both papillary and clear cell RCC have been reported.^7,11,12,13^ The clear cell RCC with a *MITF* p.E318K alteration in this study had a loss of function alteration of the *VHL* gene, while the papillary RCC reported by Lang et al^11^ exhibited gains of chromosomes 7 and 17, both molecular hallmarks of the underlying tumor types. This finding argues against unique genotype-phenotype associations for this alteration as well. In contrast, most patients with BHD syndrome present with tumors that have unique morphologic features that resemble both oncocytoma and chromophobe RCC (hybrid oncocytic tumors).^14,15^ Rare cases of clear cell, papillary and undifferentiated RCC have been reported in BHD; however, it is unclear whether these are sporadic tumors.^14,15^ For the most part, patients with BHD syndrome appear to have a unique genotype-(histologic) phenotype association with hybrid-oncocytic tumors. In addition, uveal melanoma and mesothelioma represent tumor types that have been reported in only rare instances for patients with BHD syndrome; this finding highlights the importance of germline testing in patients with multiple tumors.

### Limitations

This study has limitations. Some limitations of our study include IHC and medical record review-based screening for *BAP1* alterations. It is possible that patients with missense *BAP1* alterations may have retained expression by IHC and were therefore missed. With regard to *MITF* p.E318K alterations, only clear cell RCCs were screened and non–clear cell RCCs with this alteration may have been missed. In addition, this study was not designed to assess the prevalence of *FLCN* and non–*BAP1*/ *MITF* p.E318K alterations, and the true prevalence of all germline alterations may have been underestimated. Although the composition of patients included within the institutional nephrectomy registry is not biased toward patients with more indolent/aggressive disease, findings pertaining to the prevalence of melanoma and/or mesothelioma and renal neoplasia within this cohort require independent validation in larger data sets.

## Conclusions

Current National Comprehensive Cancer Network criteria for evaluation of hereditary RCC predisposition syndromes recommend testing for all individuals who meet the following criteria: RCC at less than or equal to age 46 years, bilateral/multifocal tumors, first-degree or second-degree relatives with RCC or with a known pathogenic/likely pathogenic variant in cancer susceptibility genes and specific histologic features associated with certain renal neoplasia. Four of 5 patients in our study met these criteria and our results support the continued use of these guidelines. However, given the association of hereditary predisposition syndromes in patients with melanoma and/or mesothelioma with renal neoplasia, universal screening may be warranted in this patient population with a panel that includes *BAP1*, *MITF*, and *FLCN*.

## Notes

Supplement. **eFigure.** Histologic Subtypes of Renal Neoplasia Associated With Melanoma/Mesothelioma
