# Supplementary material for: Assessment of Risk of Hereditary Predisposition in Patients With Melanoma and/or Mesothelioma and Renal Neoplasia
Source: JAMA Netw Open. 2021 Nov 12;4(11):e2132615. doi: 10.1001/jamanetworkopen.2021.32615 (PMC8590170; doi:10.1001/jamanetworkopen.2021.32615)
Supplement: Supplement. — eFigure. Histologic Subtypes of Renal Neoplasia Associated With Melanoma/Mesothelioma [file jamanetwopen-e2132615-s001.pdf]

Supplemental Online Content

Gupta S, Erickson LA, Lohse CM, et al. Assessment of risk of hereditary predisposition in patients with melanoma and/or mesothelioma and renal neoplasia. JAMA Netw Open. 2021;4(11):e2132615. doi:10.1001/jamanetworkopen.2021.32615

eFigure. Histologic Subtypes of Renal Neoplasia Associated With Melanoma/Mesothelioma.

This supplemental material has been provided by the authors to give readers additional information about their work.

© 2021 Gupta S et al. *JAMA Network Open*.

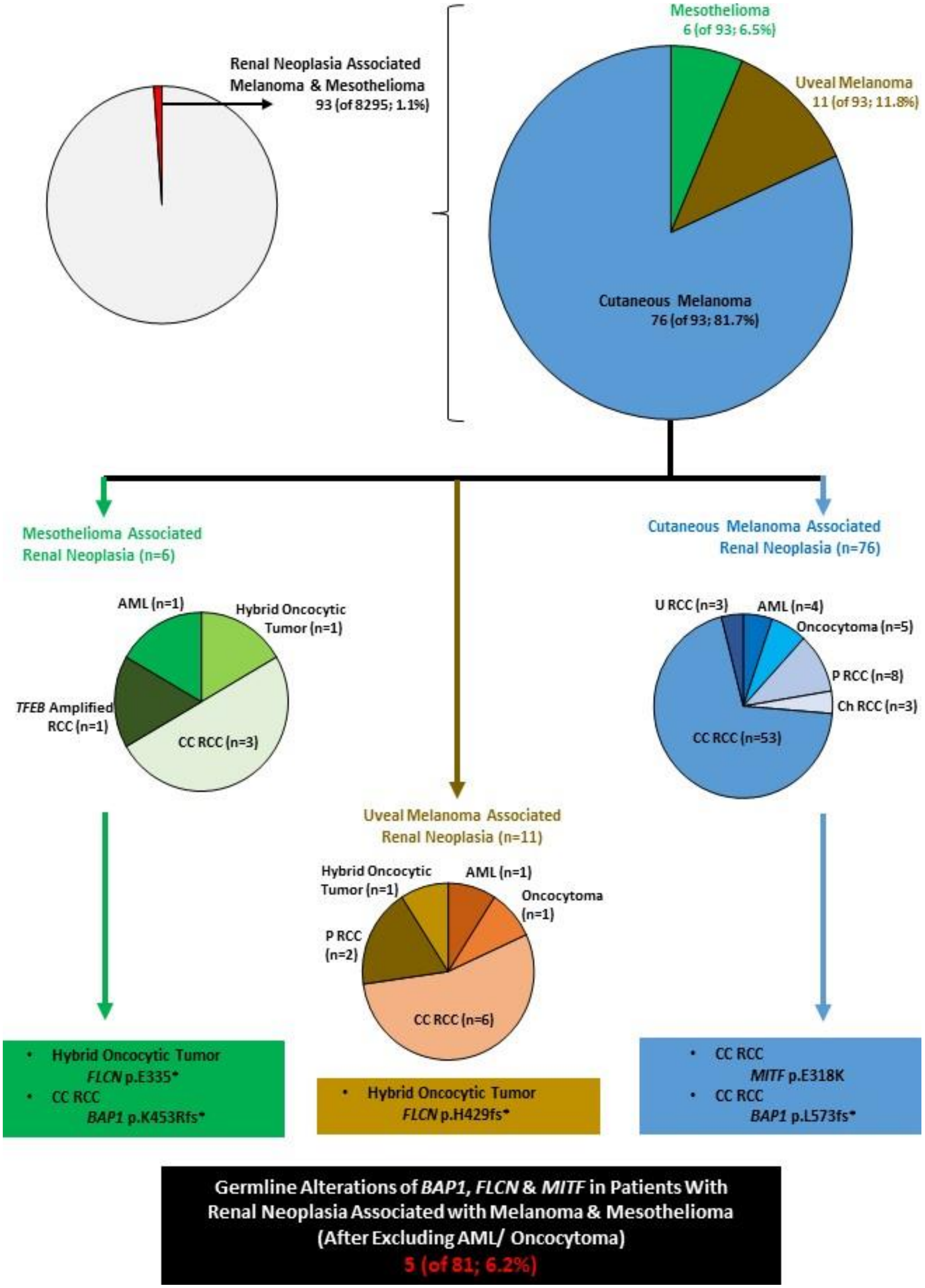

eFigure. **Histologic subtypes of renal neoplasia associated with melanoma/ mesothelioma.** A schematic representation of the prevalence of histologic subtypes of renal neoplasia associated with cutaneous/uveal melanoma and mesothelioma has been depicted. Likely germline alterations for the *BAP1*, *FLCN* and *MITF* genes have been shown. AML: angiomyolipoma; RCC: renal cell carcinoma; CC RCC: clear cell RCC; Ch RCC: chromophobe RCC; P RCC: papillary RCC; U RCC: unclassified RCC.

© 2021 Gupta S et al. *JAMA Network Open*.
